# Using detergent-enhanced LAMP for African trypanosome detection in human cerebrospinal fluid and implications for disease staging

**DOI:** 10.1371/journal.pntd.0007631

**Published:** 2019-08-19

**Authors:** Dennis J. Grab, Olga V. Nikolskaia, Bertrand Courtioux, Oriel M. M. Thekisoe, Stefan Magez, Maxim Bogorad, J. Stephen Dumler, Sylvie Bisser

**Affiliations:** 1 Department of Pathology, Uniformed Services University of the Health Sciences, Bethesda, Maryland, United States of America; 2 Department of Pathology, Johns Hopkins University School of Medicine, Baltimore, Maryland, United States of America; 3 Institute of Neuroepidemiology and Tropical Neurology, School of Medicine, CNRS FR 3503 GEIST, University of Limoges, INSERM UMR1094 Tropical Neuroepidemiology, Limoges, France; 4 Unit for Environmental Sciences and Management, North-West University, Potchefstroom, South Africa; 5 Laboratory for Biomedical Research, Ghent University Global Campus, Incheon, South Korea; 6 Laboratory for Cellular and Molecular Immunology, Vrije Universiteit Brussel, Brussels, Belgium; 7 Pasteur Institute, Cayenne, French Guiana, France; Foundation for Innovative New Diagnostics (FIND), SWITZERLAND

## Abstract

**Objective:**

Where human African trypanosomiasis (HAT) patients are seen, failure to microscopically diagnose infections by *Trypanosoma brucei gambiense* in blood smears and/or cerebrospinal fluid (CSF) in the critical early stages of the disease is the single most important factor in treatment failure, a result of delayed treatment onset or its absence. We hypothesized that the enhanced sensitivity of detergent-enhanced loop-mediated isothermal amplification (LAMP) will allow for point of care (POC) detection of African trypanosomes in the CSF of HAT patients where the probability for detecting a single parasite or parasite DNA molecule in 1 μL of CSF sample is negligible by current methods.

**Methodology:**

We used LAMP targeting the multicopy pan-*T*. *brucei* repetitive insertion mobile element (RIME LAMP) and the *Trypanosoma brucei gambiense* 5.8S rRNA-internal transcribed spacer 2 gene (TBG1 LAMP). We tested 1 μL out of 20 μL sham or Triton X-100 treated CSFs from 73 stage-1 and 77 stage-2 HAT patients from the Central African Republic and 100 CSF negative controls.

**Results:**

Under sham conditions, parasite DNA was detected by RIME and TBG1 LAMP in 1.4% of the stage-1 and stage-2 gambiense HAT CSF samples tested. After sample incubation with detergent, the number of LAMP parasite positive stage-2 CSF’s increased to 26%, a value which included the 2 of the 4 CSF samples where trypanosomes were identified microscopically. Unexpected was the 41% increase in parasite positive stage-1 CSF’s detected by LAMP. Cohen’s kappa coefficients for RIME versus TBG1 LAMP of 0.92 (95%CI: 0.82–1.00) for stage-1 and 0.90 (95%CI: 0.80–1.00) for stage-2 reflected a high level of agreement between the data sets indicating that the results were not due to amplicon contamination, data confirmed in χ^2^ tests (p<0.001) and Fisher’s exact probability test (p = 4.7e^-13^).

**Conclusion:**

This study detected genomic trypanosome DNA in the CSF independent of the HAT stage and may be consistent with early CNS entry and other scenarios that identify critical knowledge gaps for future studies. Detergent-enhanced LAMP could be applicable for non-invasive African trypanosome detection in human skin and saliva or as an epidemiologic tool for the determination of human (or animal) African trypanosome prevalence in areas where chronically low parasitemias are present.

## Author summary

Human African trypanosomiasis is a fatal disease (if untreated) spread by bloodsucking tsetse flies. These protozoan parasites first enter the lymph and blood to invade many organ systems (early stage sleeping sickness). Weeks to months later, the parasites invade the brain causing a wide variety of neurological symptoms (late stage sleeping sickness). In rural clinical settings, diagnosis still relies on the detection of these microbes in blood and cerebrospinal fluid (CSF) by microscopy. LAMP, or loop-mediated isothermal amplification of DNA, is a technique that can specifically detect very small amounts of DNA from an organism. We previously showed that by simply adding detergent during sample preparation, the analytical sensitivity of LAMP targeting many gene copies is greatly improved, presumably because DNA is released from the pathogen cells and dispersed through the sample. We demonstrated proof of principle using pathogenic trypanosomes in different human body fluids (CSF or blood) and showed that this simple modification should be applicable for diagnosis of other microbial infections where cells are sensitive to detergent lysis. After completion of the above published study, we tested a collection of clinical CSF samples from African patients diagnosed with early or late stage sleeping sickness based on current World Health Organization (WHO) guidelines. For proof-of-concept we tested only a single microliter of detergent-treated CSF to test for late stage disease. We predicted that a significant number of the late stage samples would be LAMP positive, while the early stage CSFs would yield predominantly negative results. Instead, our study detected trypanosome DNA in patient CSF independent of African sleeping sickness stage, results that may be consistent with early brain entry and other scenarios that identify critical knowledge gaps for future studies.

## Introduction

In East Africa, the tsetse fly-transmitted protozoan parasite *Trypanosoma brucei rhodesiense* causes acute human African trypanosomiasis (HAT/ sleeping sickness) [1]. Transmitted from animals to man, *T*. *b*. *rhodesiense* infection is a zoonosis characterized by relatively high parasite loads. Over 97% of all HAT cases occur in West and Central Africa where the disease is caused by *T*. *b*. *gambiense*, which causes chronic disease with intermittent parasitemias characterized by low parasite numbers [2, 3]. In 2016, the number of patients with HAT reported by the World Health Organization (WHO) was fewer than 2,000; however, with many unreported cases, the estimate of actual number of infected people in the remaining endemic countries in Africa is probably higher [4]. History has shown that HAT reappears at epidemic cyclic intervals as parasites from chronically infected individuals without clinical signs of disease or harbored in animal reservoirs re-emerge back into the population [5–7].

The disease is marked by an early systemic hemolymphatic stage-1 phase, where the clinical symptoms and signs are easily confused with those of other infectious diseases (i.e. malaria, viral syndromes). Left untreated, the parasites invade the central nervous system (CNS; stage-2), a process that usually takes weeks to months with *T*. *b*. *rhodesiense* or months to years for *T*. *b*. *gambiense* infections. Both parasites cause white matter encephalitis (leukoencephalitis) that belies neuropathologic manifestations that lead to death if untreated [reviewed in [8, 9]]. Night time insomnia and day time drowsiness, which give the disease its name, are the most characteristic neurologic signs of gambiense HAT. However, the somnolence and other late stage mental signs are less common in Rhodesian disease, although there may be mental slowness or dullness and drowsiness or coma in terminal disease [10]. A key issue in the diagnosis and treatment of HAT is to distinguish reliably CNS involvement with HAT from the early stage disease. Accurate staging of HAT is critical because failure to treat a patient with CNS involvement using stage-2 drugs will lead inevitably to death from the disease, yet inappropriate CNS treatment in an early-stage patient carries a high risk of unnecessary drug toxicity and potentially death.

The diagnosis and staging of HAT in the rural clinical setting where most patients are found, is time consuming, difficult and still relies largely on the microscopic detection of parasites in clinical samples (blood smear, lymph, CSF). While this approach is inherently insensitive, it is still considered the unofficial gold-standard for specific diagnosis [11–13]. Where the disease is hyperendemic and since trypanosomes can be difficult to detect in CNS HAT, especially in the late stage [14], failure by these methods to correctly classify stage-2 from stage-1 disease is probably the single most important contributor to disease progression and treatment failure [11]. While *T*. *b*. *rhodesiense* detection in blood is frequently successful, for *T*. *b*. *gambiense* infections, where only a few parasites are present in the peripheral circulation or in CSF, a thorough search is required, but is time consuming and subjective. Concentration techniques such as double centrifugation or mini-anion exchange columns (mAECT) are usually necessary [11–13].

Because of the inherent difficulties associated in detection of parasites in gambiense HAT patient CSF samples, examination of white cell count/protein concentrations suggestive of chronic meningoencephalitis is required. Because CNS involvement is often-silent, staging relies on lumbar puncture to assess chronic meningoencephalitis, especially in field screening wherein few cases have neurological signs [14, 15]. CSF leukocyte counts are scored according to stage-2 cut-offs recommended by WHO [12]. Detection of trypanosomes in CSF does not define ‘chronic’ CNS infection, since the immune system may also destroy the parasite [14, 16]. Hence, determination of persistent disease to eliminate parasite reservoirs in the population remains an unmet challenge.

Assay sensitivity for *T*. *b*. *gambiense* detection even by molecular tests, i.e. polymerase chain reaction (PCR) and loop-mediated isothermal amplification (LAMP) [17–23], is often limited by the stoichiometric presence of the parasite in the assayed sample. Cox *et al*. [24] reported the difficulty to establish true trypanosome (*T*. *congolense*, *T*. *vivax*, *T*. *b*. *brucei*) prevalence in blood spotted on paper cards using specific PCR detection tools in indigenous African zebu cattle due to chronically low parasitemias [24]. Because parasite DNA was unevenly distributed across the card, a single punch from an FTA card was insufficient to confirm infectivity: i,e. the stochastic sampling effect results in underestimation of prevalence [24]. The same stoichiometric apply to blood/CSF-based molecular assays sufficiently sensitive to detect DNA below the content of a single parasite; i.e. the detection limit of the assay is still restricted by the number of parasites present in the volume of sample assayed [22].

Remarkably, the answer was simple: i.e. LAMP assays that recognize multi-copy gene targets for trypanosome DNA are dramatically enhanced by sample pretreatment with detergents to lyse or solubilize their DNA prior to assay [22]. By pre-lysing cells with detergent before application of parasite-specific LAMP primers and amplification that recognize multi-copy gene targets we have been able to markedly improve the detection of parasite genomic DNA by LAMP. Using human CSF spiked with trypanosomes as direct source of DNA template, we found that detergent-enhanced LAMP assay targeting multi-copy trypanosome genes reached analytical sensitivities about 100 to 1000-fold or lower [22]. Similar increases in LAMP assay analytical sensitivity were also found using DNA extracted from filter paper cards containing blood pretreated with detergent before card spotting, or using DNA extracted from blood samples spotted on detergent-pretreated air-dried cards for improved assay reproducibility [22]. Hayashida et al [25] later showed that RNA could also be amplified directly from detergent-lysed blood samples.

Here we assess and show that detergent-enhanced LAMP is a simple point of care (POC) molecular assay platform for *T*. *b*. *gambiense* parasite detection in clinical samples where chronically low parasitemias are expected. As stage determination relies on lumbar puncture to examine CSF for trypanosomes confirming neurological invasion [15], our findings are intriguing in that the data provide evidence for detection of genomic *T*. *b*. *gambiense* DNA in the CSF independent of HAT stage. Overall, it is predicted that this simple technological advance will greatly improve POC pathogen detection including those trypanosomes in the so-called aparasitemic individuals who also may or may not be seropositive for the parasites and environmental monitoring.

## Materials and methods

### The clinical samples and ethical statement

A de-identified cohort of 150 clinical samples was obtained from HAT patients during studies in Central African Republic (Batangafo focus, 2001) under the direction of “Programme National de Lutte contre la Trypanosomose Humaine Africaine” (PNLTHA) (**S1 Table**). Written informed consent was received from these subjects prior to enrollment and/or from their parents or guardians for participants below 18 years of age. All patients in the collection were screened for clinical signs and specifically for neurological and psychiatric disturbances. Samples from patients testing positive for microscopic presence of malaria (blood smear), filariasis (blood examination by capillary tube centrifugation), schistosomiasis (when blood was detected in urine), as well as by retrospective testing of stored samples for HIV and syphilis, were excluded. All clinical samples that remained after the above clinical diagnostic procedures were aliquoted and stored in liquid nitrogen before being transported on dry ice to the Institute of Tropical Neurology at Limoges University. The anonymized samples were archived and stored at -70°C and at no time was there a break in the cold-chain until the aliquoted samples were used for the LAMP assay for this study. Because it is not ethical to perform a lumbar puncture on a healthy person, we used negative control human CSF obtained as discarded de-identified clinical samples from The Johns Hopkins Hospital Microbiology laboratory that were obtained from patients with neurological manifestations with approval of the Johns Hopkins Medicine Institutional Review Board (IRB).

### Screening for trypanosomes and HAT staging

*Gambiense* HAT was confirmed by a positive card agglutination test for trypanosomiasis (CATT) and with trypanosomes microscopically identified in blood and posterior cervical lymph nodes if latter were enlarged, and on CSF smears based on WHO guidelines adapted for populations where the prevalence of the HAT is high- (> 1%) areas [12]. Based on the prevalence of HAT in the area our cohort was obtained, our decision pathways were based on a published algorithm (Fig 1 in ref [26]) modified with a focus on having a real "stage-1" group. We considered all patients with presence of trypanosomes in blood or lymph and with less than 5 white blood cells (WBC)/μL), no trypanosomes in CSF and no neurological signs as stage-1. All others at stage-2, so patients with 10 cells without trypanosomes in CSF were considered stage-2. A positive CATT at ≥1:16 dilution with documented trypanosomes (either by microscopy or by mAECT) was our reference standard for confirmed gambiense HAT. Positive CATT ≥1:16 without evidence of trypanosomes was considered serologic HAT. Positive CATT ≥1:4 but <1:16 without evidence of trypanosomes was considered possible HAT. Negative CATT (<1:4) was considered not HAT (control). Patients with HAT and CSF with evidence of IgM (nephelometric detection) [27], or trypanosomes (microscopic detection), or increased CSF white cell blood count (> 5 WBC/μL), or relapse after stage-1 HAT treatment, were classified as stage-2 HAT. Patients presenting with HAT without IgM or trypanosomes in the CSF and with CSF cell counts ≤5 WBC/μL who had not relapsed after stage-1 treatment were also considered stage-1 HAT. The presence or absence of trypanosomes (by microscopy or mAECT) dictated whether stage-1 and stage-2 HAT cases were confirmed or serologic, respectively. Overall, the final cohort consisted of de-identified archived CSFs from 150 HAT patients—includes 95 adults; 21 patients between the ages of 12 to 17; and 34 patients <12 years of age—clinically defined as stage-1 (73 samples) or stage-2 (77 samples). Negative control samples were available from 100 patients.

### RIME and TBG1 LAMP primer sets

Two LAMP primer sets targeting the pan-*T*. *brucei*—500 copy—repetitive insertion mobile element (RIME) of subgenus *Trypanozoon* (GenBank Accession No. K01801) (RIME LAMP) and the—200 copy—*T*. *b*. *gambiense* 5.8S rRNA-internal transcribed spacer 2 (5.8S-ITS2) gene (GenBank Accession No. AF306777) (TBG1 LAMP) were used (**S2 Table**). The analytical specificity for trypanosome DNA and sensitivity (equivalent to 0.01 parasite or less) for RIME and TBG1 LAMP are well documented [20, 22, 23, 28, 29]. All LAMP primers (Forward and Backward Primers F3 and B3; Forward and Backward Inner Primers FIP and BIP; and Forward and Backward Loop Primers LF and LB) were synthesized and HPLC-purified by Integrated DNA Technologies (IDT).

### LAMP reaction assays

A 10% (w/v) Triton X-100 stock solution was made by adding 1 g Triton X-100 to a final volume of 10 mL DNase/RNase free water (Qiagen). The clinical CSF samples were adjusted to contain 1/20 volume of 10% Triton X-100 (final concentration 0.5% Triton), or 1/20 volume deionized water (untreated sham CSF) [22]. The CSFs were assayed immediately or after a 60 min incubation at ambient temperature to allow for complete detergent lysis prior to LAMP as we previously described [22]. All LAMP reactions using commercially available kits (Eiken Chemical Co, Japan) were previously optimized for reagent concentration, reaction time and temperature in real-time in a Loopamp real-time turbidimeter LA320C (Teramecs, Tokyo, Japan) as previously described [17, 22, 29, 30]. Briefly, the reaction contained 12.5 μL of 2x LAMP buffer (40 mM Tris-HCl [pH 8.8], 20 mM KCl, 16 mM MgSO_4_, 20 mM [NH_4_]_2_SO_4_, 0.2% Tween 20, 1.6 M Betaine, 2.8 mM of each deoxyribonucleotide triphosphate), 1.0 μL primer mix (5 pmol each of F3 and B3, 40 pmol each of FIP and BIP) or 1.3 μL when LF and LB (20 pmol each) were included, 1 μL (8 units) *Bst* DNA polymerase (New England Biolabs, Tokyo, Japan) and 1 μL of human CSF. Final volumes were adjusted to 25 μL with water. LAMP reactions monitored for 60 min by measuring turbidity in real-time as previously described [22] were conducted in duplicate and at optimal reaction temperatures, 62°C for RIME LAMP and 63°C for TBG1 LAMP, prior to termination at 80°C for 5 min. We considered precipitation occurring after a reaction time of 60 minutes to be nonspecific artifacts. For end-point analysis (**S1 Fig**) the amplified products were analyzed using the E-Gel high throughput DNA electrophoresis system with ethidium bromide or SYBR green incorporated into the gels (Invitrogen), or after addition of hydroxy naphthol blue (HNB) to monitor the sample color change from violet to sky blue, a readout unaffected by detergent, has been interpreted by independent observers as the easiest to see [23]. As with any DNA amplification method, standard precautions for avoiding template contamination [31] also apply for LAMP-based assays. Thus, the reactions were assayed in 4 blocks with each block containing 8 samples with 2 no template controls for every 6 samples assayed. A false positive response from any no template or non-HAT CSF control negated the entire 32-reaction run and the samples were re-assayed.

### Statistical analysis

Statistical significance between data sets obtained with RIME versus TBG1 LAMP was determined using the Cohen’s kappa coefficient (Vassar Stats; http://vassarstats.net/kappa.html) as a measure of agreement between Triton X-100 pretreated patient CSF samples that were positive or negative for trypanosomes. For reference, the following Kappa coefficients and levels of agreement are as follows: 0.80 to 1.00 = Very good agreement, 0.60 to 0.80 = Good agreement, 0.40–0.60 = Moderate agreement, 0.20–0.40 = Fair agreement, while values <0.20 = Poor agreement. Additionally, we applied the χ^2^ test and Fisher’s exact probability test to the 2x2 contingency table generated comparing the two approaches (Vassar Stats; http://vassarstats.net/tab2x2.html).

## Results

Using pan-*T*. *brucei* RIME LAMP as our ‘gold standard’ LAMP assay, conventional WHO staging concepts predicted that a significant number of the stage-2 CSF samples would be LAMP positive, while the stage-1 CSFs would yield predominantly negative results. To establish baseline clinical control values, RIME and TBG1 LAMP were performed on at least 100 negative control CSF samples obtained from the Johns Hopkins Hospital (Baltimore, MD USA). All control CSF samples were found to be RIME- and TBG1 LAMP-negative whether or not they were pre-incubated for 1 h with detergent prior to assay to allow for optimal sample lysis [22]. We then tested the archived CSFs from 150 HAT patients—includes adults and children the between the ages of 3 to 17 years)—clinically defined as stage-1 (73 samples) or stage-2 (77 samples of which 4 were microscopically parasite positive) (**S1 Table**). Overall, under sham conditions using only 1 μL sample and pan-*T*. *brucei* RIME LAMP, we found that 1/73 (1.4%) of the stage-1 samples tested positive possibly due to the presence of circulating free DNA (cfDNA), as the patient was previously treated for HAT (**S1B Table**; see below) and 2/77 (2.6%) of the stage-2 HAT CSF samples tested were parasite DNA-positive by pan-*T*. *brucei* RIME LAMP. We have shown that release of parasite DNA by 0.5% Triton required between 30 and 60 min incubation [22]. While the percentage of trypanosome DNA-positive CSFs by RIME LAMP conducted immediately after Triton X-100 addition increased 2-fold, LAMP conducted 60 min after detergent addition revealed that 21 out of 77 (27.3%) stage-2 CSFs tested were parasite DNA-positive (**Table 1A**). In patients with HAT, especially with *T*. *b*. *rhodesiense*, it is more difficult to detect trypanosomes in the CSF compared with the blood where the parasitaemia is generally high [9] though much less so in *T*. *b*. *gambiense*. Remarkably, even under our stringent assay conditions whereby only 1 μL of CSF was directly assayed, RIME LAMP with detergent did identify 2 (1 adult (ID 8) and 1 pediatric <12 years of age (ID 107); **S1 Table**) of the 4 patient CSFs in which trypanosomes were identified microscopically. Somewhat unexpected was the dramatic 1.4% (2/73) to 42.5% (31/73) increase in the number of parasite DNA-positive CSFs for stage-1 samples (**Table 1A**).

**Table 1:** Fraction of CSF samples LAMP positive for trypanosome DNA.

**A) For all adult and pediatric patients**					
	**RIME LAMP**			**TBG1 LAMP**	
	**Preincubation**			**Preincubation**	
**HAT stage**	**Sham** **60 min**	**Tx100** **60 min**	**Tx100** **0 min**	**Sham** **60 min**	**Tx100** **60 min**
**1**	1/73 (1.4%)	31/73 (42.5%)	2/73 (2.7%)	1/72 (1.4%)	29/73 (39.7%)
**2**	2/77 (2.6%)	21/77 (27.3%)	4/77 (5.2%)	1/76 (0%)	19/77 (24.7%)
**1+2**	3/150 (2.0%)	52/150 (34.7%)	6/150 (4.0%)	2/149 (1.4%)	47/150 (31.3%)
**B) For patients with no prior trypanocide treatment**					
	**RIME LAMP**			**TBG1 LAMP**	
	**Incubation**			**Incubation**	
**HAT stage**	**Sham** **60 min**	**Tx100** **60 min**	**Tx100** **0 min**	**Sham** **60 min**	**Tx100** **60 min**
**1**	0/64 (0%)	23/64 (35.9%)	1/64 (1.6%)	1/64 (1.6%)	20/64 (46.9%)
**2**	2/64 (3.1%)	16/64 (25.0%)	4/64 (6.3%)	1/64 (1.3%)	17/64 (26.6%)
**1+2**	2/128 (1.6%)	44/128 (34.4%)	5/128 (3.9%)	2/128 (1.6%)	37/128 (28.9%)

It has been suggested that molecular tests (PCR) may not be suitable for post-treatment follow-up of HAT cure because of persistence of trypanosome cfDNA that may lower test specificity [32–34]. Released into the bloodstream as a result of cell death, necrosis, or by release by viable cells, cfDNA has been found in many disease conditions [35]. In our cohort, 22 samples (9 stage-1 and 13 stage-2) were from patients that had previously been treated (with some trypanocide) for HAT prior to sample collection (**S1B Table**). While 8 out of 9 stage-1 and 5 out of 13 stage-2 Triton-pretreated samples were RIME LAMP positive, one stage-1 sample, ID2, and two stage-2 samples, ID1 and ID12, were strongly LAMP positive under sham condition. In fact, this was the only sham sample in the entire 150 sample cohort positive for trypanosome DNA (Compare **S1A Table** to **S1B Table**).

**Fig 1:**
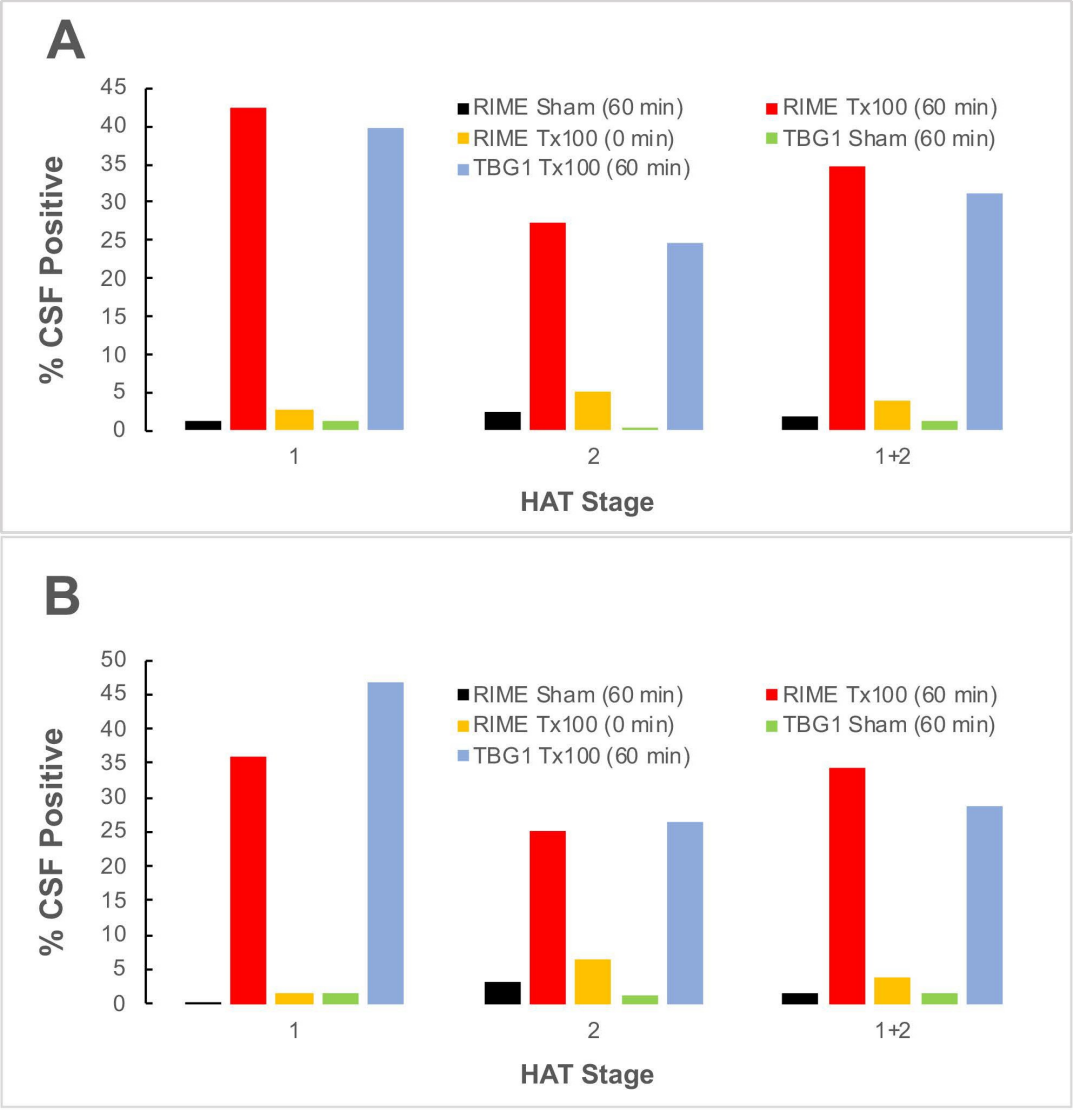
Percentage (%) of CSF samples positive for trypanosome DNA by RIME and TBG1 LAMP based on Table 1 data.

A re-analysis of the 128 samples from HAT patients who never received any trypanocide prior to collection (**S1A Table**), also showed that while none of the stage-1 samples were LAMP-positive, 3.1% of the stage-2 HAT CSF samples tested were parasite DNA-positive by RIME LAMP, a value predicted provided that the samples on average had 1 parasite / 20 μL sample assayed: i.e. 1/20 x 64 = 3.2%. RIME LAMP conducted on these Triton pretreated CSF samples showed a 25% (16/64) increase in the number stage-2 CSFs as well as a 35.9% (23/65) increase in the number of parasite DNA positive stage-1 samples (**Table 1B**). These findings were confirmed by repeating the experiments in the absence or presence of Triton using TBG1 LAMP targeting the *T*. *b*. *gambiense* specific 5.8S-ITS2 gene (**S1 Table** and **Table 1 and Fig 1**).

When properly executed, detergent-enhanced LAMP is able to detect very low parasite numbers, but the assay’s sensitivity is a potential drawback because of risk for amplicon contamination. Thus, we used the Cohen’s kappa test to compare the LAMP data from two unrelated trypanosome gene targets. Based on data derived from the entire cohort, Cohen’s kappa test identified a high level of agreement between RIME and TBG1 LAMP in samples that had been pre-incubated with Triton X-100 prior to assay (the most sensitive condition) when testing CSFs, thus indicating that the results are not due to amplicon contamination (**Table 2**). Kappa coefficients for RIME versus TBG1 LAMP of 0.92 (95%CI: 0.82–1.00) for stage-1 and 0.90 (95%CI: 0.80–1.00) for stage-2 reflect a high level of agreement between the data set, data confirmed in χ^2^ tests (p<0.001) and Fisher’s exact probability test (p = 4.7e^-13^). Inclusion of the 100 negative control CSFs strengthens the level of agreement: 0.94 (95%CI: 0.87–1.00) for stage-1 and 0.92 (95%CI: 0.83–1.00) for stage-2. Re-analysis of Kappa after removing the 22 samples from patients previously treated with trypanocide (data from **S1B Table** only) did not significantly change the kappa coefficients for RIME versus TBG1 LAMP for stage-1 and for stage-2 HAT; 0.90 (95%CI: 0.78–1.00) for stage-1, 0.88 (95%CI: 0.75–1.00) for stage-2, and 0.89 (95%CI: 0.81–0.98) for all stages.

**Table 2:** Cohen’s kappa test comparing detergent-enhanced RIME versus TBG1 LAMP.

**For all stage-1 and stage-2 HAT CSFs**			
**HAT Stage**	**# CSF Samples**	**kappa Coefficient**	**95% Confidence Interval**
**1**	73	0.92	0.82–1.00
**2**	77	0.90	0.80–1.00
**1+2**	150	0.91	0.84–1.00
**For all stage-1 and stage-2 HAT CSFs including non-HAT CSF controls**			
**HAT Stage**	**# CSF Samples**	**kappa Coefficient**	**95% Confidence Interval**
**1**	73	0.94	0.87–1.00
**2**	77	0.92	0.83–1.00
**1+2**	250	0.93	0.87–1.00

*After 60 min preincubation with Triton X-100 prior to LAMP assay

## Discussion

This paper is a continuation of a published study [22] that demonstrated the power of detergent-enhanced LAMP assay to detect low numbers of live African trypanosomes in human CSF or blood experimentally spiked with the parasites. The findings led us to predict that detergent-enhanced LAMP will provide a simple, sensitive, and specific assay platform for detection of parasites in clinical samples for *T*. *b*. *gambiense* HAT where low parasitemias are to be expected. Taking into consideration that the concentration of *T*. *b*. *gambiense* in 1 mL of HAT patient blood is often < 100 parasites (i.e. < 2 parasites/20 μl) [3], for proof-of-concept a minimal parasite detection threshold of ≥50 parasites/mL (or 1 parasite/20 μL sample) was thus established. Therefore, while one would have to test up to 20 individual LAMP assays based on a 1 uL test sample volume to be certain of detecting the DNA from the single intact parasite in the sample, detergent-enhanced RIME or TbG1 LAMP would only require a single determination. However, while any positive response would indicate that the sample had at least 50 parasites/mL, a negative response does not necessarily mean that the sample was truly negative, just below the 50 parasite/mL detection limit set by the limit of the sample size tested.

While based on a single clinical cohort our findings are intriguing in that the data provide evidence for detection of genomic *T*. *b*. *gambiense* DNA in the CSF independent of HAT stage and patient age. The data also provide suggestive clinical evidence for early CNS entry, supported by clues drawn from the *T*. *b*. *brucei* and *T*. *b*. *rhodesiense* literature [36–38]. In a murine brain invasion model, intravital microscopy revealed that African trypanosomes causing acute (i.e. *T*. *b*, *rhodesiense* IL1852 and *T*. *b*. *brucei* 90–13) and chronic (i.e. *T*. *b*. *brucei* GVR/35) disease directly invade the cerebral cortex parenchyma within hours after high dose (10^6^ parasites) retroorbital administration followed by inflammatory cell recruitment soon thereafter without associated neurological signs [36]. When given a low dose intraperitoneal inoculation of *T*. *b*. *brucei* GVR/35 (2×10^4^ parasites), brain imaging documented parasites in the meninges [37] and cerebellum days earlier than was predicted from pharmacological studies alone [39, 40]. with CNS impairment occurring prior to the onset of established stage-2 disease [41]. Neurological signs in rats can also occur before trypanosomes enter the neuropil and parasite/lymphocyte invasion of brain parenchyma [42], which appears to be in line with a clinical report that the early neurological features in some rhodesiense HAT patients with stage-1 HAT can be detected prior to the onset of established stage-2 disease [43]. Finally, testing 5 different algorithms used by Me´decins Sans Frontie`res HAT programmes Checchi et al [26] found that while stage 2 classification was highly accurate, about one third of stage 1 cases were misclassified as stage 2. Thus, these [26] and current WHO criteria leave open the need to consider other approaches that go beyond the prevailing notion that the transition from acute to CNS stage disease entails crossing brain barriers to enter the CNS to initiate inflammation that define clinical staging for CNS [41].

The development of simple non-invasive detergent-enhanced LAMP assay platforms for POC detection of trypanosomes in human saliva and skin with sensitivities equivalent or superior to current invasive methods, is an important POC diagnostic goal. Lejon *et al*. first showed that detection of *T*. *b*. *gambiense* antigens in a non-invasive saliva-based HAT test format was theoretically possible [44, 45]. A 180-day study in non-human primates comparing LAMP and PCR targeting the single copy TgsGP gene, found TgsGP LAMP a more sensitive assay format for detecting *T*. *b*. *gambiense* DNA in several biofluids including serum and saliva [46]. Up to 77 days post infection (dpi) TgsGP LAMP recorded 100% detection in the saliva samples [46]. While LAMP recorded 33% detection in the serum 180 dpi, TgsGP LAMP failed to detect trypanosomes in saliva after 140 days. Replacing TgsGP-LAMP with detergent enhanced RIME-LAMP or TBG1 LAMP (this paper and Ref [22]) would in theory yield a 2-log more sensitive diagnostic.

The same concepts could also be applied for detection of human skin [47, 48] and subcutaneous adipose tissue [49–51] dwelling trypanosomes in HAT patients [47, 48]. Capewell *et al*. [47] reported that about 0.5% of more than 1000 skin biopsies collected for a river blindness study in the Democratic Republic of the Congo had morphologically identified skin-dwelling *T*. *b*. *gambiense* in extravascular and subcutaneous adipose tissue a finding that could explain the persistence of HAT in some areas despite eradication efforts [47]. The skin would provide a large reservoir for the parasites; a consideration for identifying clinically seropositive but aparasitemic individuals. One caveat is that while the trypanosomes appear to infect patches of the skin, parasites in other skin areas may be harder to find. We predict that DNA released from even a single parasite solubilized by detergent will be sufficient for LAMP amplification. Overall, the skin is another theoretical assay site where non-invasive detergent enhanced LAMP could have a potential diagnostic impact.

### Conclusion

Overall, the evidence support the idea that current WHO criteria need to go beyond the prevailing notion that the transition from acute to CNS stage disease entails crossing brain barriers to enter the CNS to initiate inflammation that define clinical staging for CNS [41]. Whether detection of trypanosome genomic DNA by detergent-enhanced LAMP in the CSF independent of HAT stage provides supportive clinical evidence for early CNS entry, reflects parasite contamination of CSF with infected blood during lumbar puncture, and/or other scenarios, identify critical knowledge gaps for future studies. Controlled laboratory and field validation of detergent LAMP [22] or the adaptation of more recent ultrasensitive LAMP assays based on DNA-protein chimeras called LAMPoles for detection of non-nucleic based targets [52] that include HAT staging biomarker molecules [53, 54] that take into account saliva, skin and subcutaneous fat-dwelling trypanosomes may help facilitate development of better algorithms for HAT diagnosis and monitoring. Further, the detection of parasite DNA regardless of sample source by detergent LAMP may help provide for very early *T*. *b*. *gambiense* HAT diagnosis regardless of staging. The use of these LAMP-based platforms as epidemiologic tools for the determination human African trypanosome prevalence in areas where chronically low parasitemias are present or in areas where there are high numbers of asymptomatic (carriers) patients should also be considered.

## Supporting information

S1 Fig End point RIME LAMP. For the examples shown, HAT stage-1 (ID 2, 19), HAT stage-2 (ID 1, 10), negative control human CSF and trypanosome DNA (positive control) were preincubated with Triton X-100 or water (sham). One μl was removed for trypanosome DNA detection by RIME LAMP in real-time (see **S1 Table**) and by end point visual analysis: i.e. turbidity, HNB color change from violet to sky blue, and on 2% agarose gels stained with ethidium bromide. **Panel A** shows the results for RIME LAMP on the sham pretreated clinical samples. The results for RIME LAMP on the detergent pretreated clinical samples and on the positive and negative control samples, are shown in **Panels B** and **C**, respectively.
(TIF)

S1 Table HAT staging and LAMP detection of trypanosome DNA in CSF. (DOCX)

S2 Table The LAMP primer sets. (DOCX)
