# Supplementary figures and images for: Using detergent-enhanced LAMP for African trypanosome detection in human cerebrospinal fluid and implications for disease staging
Source: PLoS Negl Trop Dis. 2019 Aug 19;13(8):e0007631. doi: 10.1371/journal.pntd.0007631 (PMC6715242; doi:10.1371/journal.pntd.0007631)

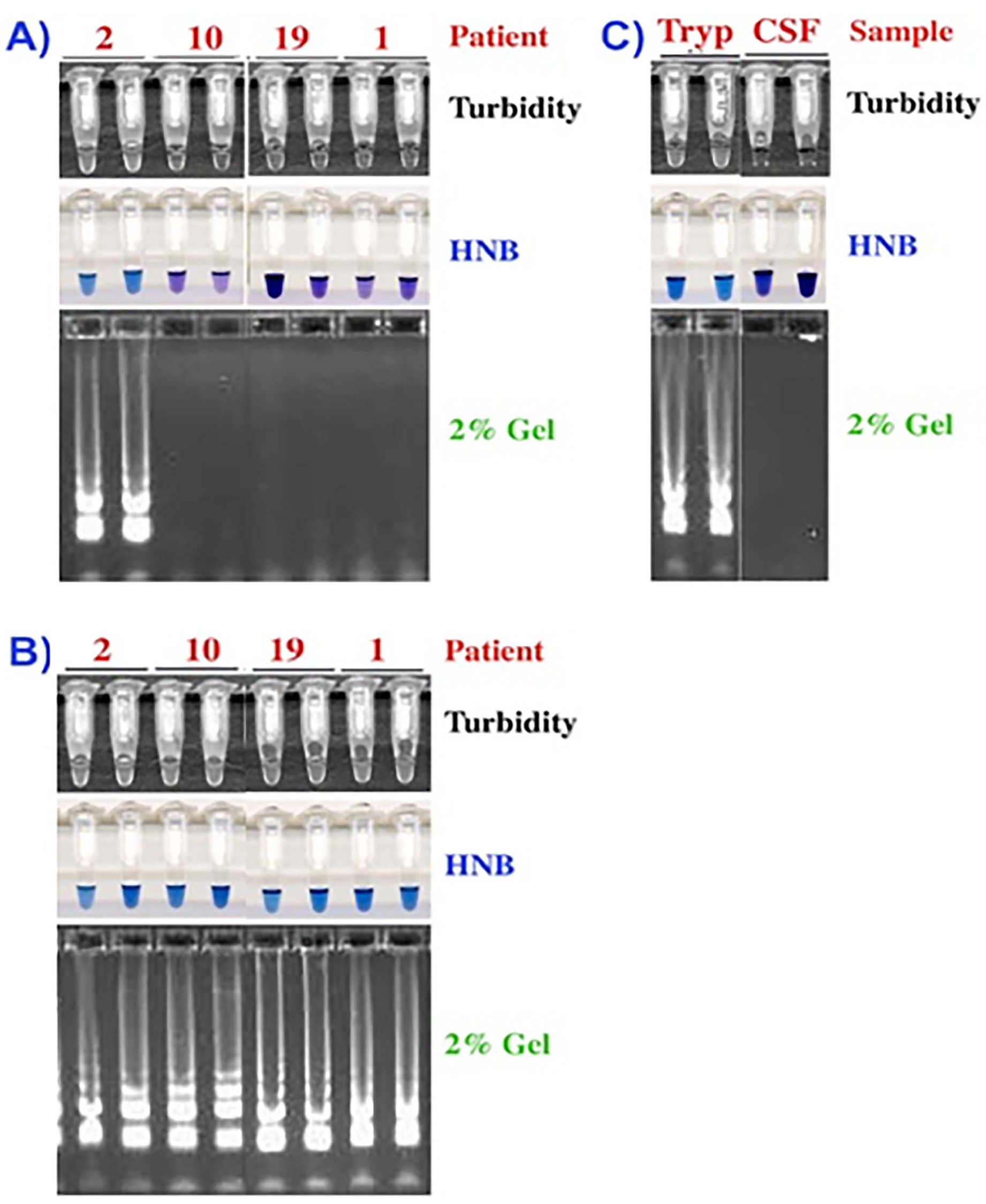

Supplement: S1 Fig — For the examples shown, HAT stage-1 (ID 2, 19), HAT stage-2 (ID 1, 10), negative control human CSF and trypanosome DNA (positive control) were preincubated with Triton X-100 or water (sham). One μl was removed for trypanosome DNA detection by RIME LAMP in real-time (see S1 Table) and by end point visual analysis: i.e. turbidity, HNB color change from violet to sky blue, and on 2% agarose gels stained with ethidium bromide. Panel A shows the results for RIME LAMP on the sham pretreated clinical samples. The results for RIME LAMP on the detergent pretreated clinical samples and on the positive and negative control samples, are shown in Panels B and C, respectively. (TIF) [file pntd.0007631.s001.tif]
