# Supplementary material for: Using detergent-enhanced LAMP for African trypanosome detection in human cerebrospinal fluid and implications for disease staging
Source: PLoS Negl Trop Dis. 2019 Aug 19;13(8):e0007631. doi: 10.1371/journal.pntd.0007631 (PMC6715242; doi:10.1371/journal.pntd.0007631)
Supplement: S1 Table — (DOCX) [file pntd.0007631.s002.docx]

| **S1 TABLE; HAT staging and LAMP detection of trypanosome DNA in CSF** | | | | | | | | | | | |
| --- | --- | --- | --- | --- | --- | --- | --- | --- | --- | --- | --- |
| **A: Data for patients who never received trypanocides prior to sample collection** | | | | | | | | | | | |
| **Patient** | **Serology** | | | | | **Stage** | **LAMP Assay Results*** | | | | |
|  | **CATT Positive** | **Trypanosomes in** | | | **Number**  **of cells**  **In CSF** |  | **RIME LAMP** | | | **TBG1 LAMP** | |
|  |  |  |  |  |  |  | **Preincubation** | | | **Preincubation** | |
|  |  | **Blood** | **Lymph** | **CSF** |  |  | **Sham**  **60 min** | **Tx100**  **60 min** | **Tx100**  **0 min** | **Sham**  **60 min** | **Tx100**  **60 min** |
| **Adults** | | | | | | | | | | | |
| 9 | yes | yes | yes | no | 4 | **1** | 0 | 0 | 0 | 0 | 0 |
| 19 | yes | yes | no | no | 3 | **1** | 0 | 2 | 0 | 0 | 2 |
| 24 | yes | yes | yes | no | 4 | **1** | 0 | 0 | 0 | 0 | 0 |
| 28 | yes | yes | yes | no | 2 | **1** | 0 | 2 | 0 | 0 | 2 |
| 29 | yes | yes | yes | no | 4 | **1** | 0 | 2 | 0 | 0 | 0 |
| 30 | yes | yes | yes | no | 2 | **1** | 0 | 2 | 0 | 0 | 2 |
| 31 | yes | yes | yes | no | 3 | **1** | 0 | 0 | 0 | 0 | 0 |
| 32 | yes | yes | no | no | 2 | **1** | 0 | 0 | 0 | 0 | 0 |
| 33 | yes | yes | no | no | 0 | **1** | 0 | 0 | 0 | 0 | 0 |
| 35 | yes | yes | no | no | 0 | **1** | 0 | 0 | 0 | 0 | 0 |
| 36 | yes | yes | yes | no | 4 | **1** | 0 | 0 | 0 | 0 | 0 |
| 37 | yes | yes | yes | no | 2 | **1** | 0 | 0 | 0 | 0 | 0 |
| 44 | yes | yes | yes | no | 0 | **1** | 0 | 0 | 0 | 0 | 0 |
| 46 | yes | yes | no | no | 1 | **1** | 0 | 0 | 0 | 0 | 0 |
| 51 | yes | yes | no | no | 4 | **1** | 0 | 2 | 2 | 1 | 0 |
| 53 | yes | yes | yes | no | 4 | **1** | 0 | 2 | 0 | 0 | 2 |
| 60 | yes | yes | no |  | 1 | **1** | 0 | 1 | 0 | 0 | 1 |
| 64 | yes | yes | yes |  | 2 | **1** | 0 | 1 | 0 | 0 | 0 |
| 67 | yes | yes | yes |  | 0 | **1** | 0 | 0 | 0 | 0 | 0 |
| 74 | yes | yes | yes |  | 2 | **1** | 0 | 0 | 0 | 0 | 0 |
| 75 | yes | yes | no |  | 0 | **1** | 0 | 0 | 0 | 0 | 0 |
| 76 | yes | yes | yes |  | 1 | **1** | 0 | 2 | 0 | 0 | 2 |
| 91 | yes | yes | no |  | 1 | **1** | 0 | 1 | 0 | 0 | 1 |
| 94 | yes | yes | yes |  | 4 | **1** | 0 | 0 | 0 | 0 | 0 |
| 100 | yes | yes | no |  | 4 | **1** | 0 | 0 | 0 | 0 | 0 |
| 112 | yes | yes | yes |  | 3 | **1** | 0 | 2 | 0 | 0 | 2 |
| 116 | yes | yes | no |  | 2 | **1** | 0 | 0 | 0 | 0 | 0 |
| 119 | yes | yes | no |  | 2 | **1** | 0 | 2 | 0 | 0 | 2 |
| 120 | yes | yes | no |  | 4 | **1** | 0 | 0 | 0 | 0 | 0 |
| 123 | yes | yes | yes |  | 3 | **1** | 0 | 0 | 0 | 0 | 0 |
| 130 | yes | yes | yes |  | 4 | **1** | 0 | 0 | 0 | 0 | 0 |
| 131 | yes | yes | no |  | 2 | **1** | 0 | 0 | 0 | 0 | 0 |
| 135 | yes | yes | yes |  | 0 | **1** | 0 | 1 | 0 | 0 | 1 |
| 140 | yes | yes | yes |  | 1 | **1** | 0 | 0 | 0 | 0 | 0 |
| 150 | yes | yes | no |  | 4 | **1** | 0 | 2 | 0 | 0 | 2 |
| 159 | yes | yes | yes |  | 4 | **1** | 0 | 0 | 0 | 0 | 0 |
|  | | | | | | | | | | | |
| 1 | yes | yes | yes | yes |  | **2** | 2 | 2 | 2 | 0 | 2 |
| 4 | yes | yes | yes | no | 15 | **2** | 0 | 1 | 1 | 0 | 1 |
| 7 | yes | yes | yes | no | 120 | **2** | 0 | 0 | 0 | 0 | 0 |
| 8 | yes | yes | yes | yes | 177 | **2** | 0 | 0 | 0 | 0 | 0 |
| 11 | yes | yes | yes | no | 11 | **2** | 0 | 0 | 0 | 0 | 0 |
| 12 | yes | yes | yes | no | 5 | **2** | 1 | 1 | 1 | 0 | 0 |
| 16 | yes | yes | no | no | 31 | **2** | 0 | 0 | 0 | 0 | 0 |
| 18 | yes | yes | yes | no | 65 | **2** | 0 | 2 | 0 | 0 | 2 |
| 27 | yes | yes | yes | no | 61 | **2** | 0 | 0 | 0 | 0 | 0 |
| 40 | yes | yes | no | no | 13 | **2** | 0 | 2 | 0 | 0 | 2 |
| 41 | yes | yes | yes | no | 20 | **2** | 0 | 1 | 0 | 0 | 1 |
| 42 | yes | yes | yes | no | 13 | **2** | 0 | 0 | 0 | 0 | 0 |
| 49 | yes | yes | yes | no | 26 | **2** | 0 | 0 | 0 | 0 | 0 |
| 50 | yes | yes | no | no | 22 | **2** | 0 | 0 | 0 | 0 | 0 |
| 59 | yes | yes | no |  | 12 | **2** | 0 | 0 | 0 | 0 | 0 |
| 68 | yes | yes | no |  | 55 | **2** | 0 | 0 | 0 | 0 | 0 |
| 69 | yes | yes | yes |  | 5 | **2** | 0 | 0 | 0 | 0 | 0 |
| 88 | yes | yes | no |  | 154 | **2** | 0 | 0 | 0 | 0 | 0 |
| 97 | yes | yes | no |  | 8 | **2** | 0 | 0 | 0 | 0 | 0 |
| 98 | yes | yes | yes |  | 22 | **2** | 0 | 0 | 0 | 0 | 0 |
| 109 | yes | yes | no |  | 8 | **2** | 0 | 0 | 0 | 0 | 0 |
| 110 | yes | yes | yes |  | 5 | **2** | 0 | 0 | 0 | 0 | 0 |
| 111 | yes | yes | yes |  | 31 | **2** | 0 | 0 | 0 | 0 | 0 |
| 114 | yes | yes | yes |  | 5 | **2** | 0 | 0 | 0 | 0 | 0 |
| 115 | yes | yes | yes |  | 5 | **2** | 0 | 2 | 0 | 0 | 2 |
| 127 | yes | yes | no |  | 20 | **2** | 0 | 0 | 0 | 0 | 0 |
| 141 | yes | yes | no |  | 16 | **2** | 0 | 1 | 0 | 0 | 1 |
| 142 | yes | yes | no |  | 12 | **2** | 0 | 0 | 0 | 0 | 0 |
| 124 | yes | yes | yes |  | 31 | **2** | 0 | 0 | 0 | 0 | 0 |
| 145 | yes | yes | yes |  | 100 | **2** | 0 | 0 | 0 | 0 | 0 |
| 147 | yes | yes | no |  | 11 | **2** | 0 | 2 | 0 | 0 | 2 |
| 152 | yes | yes | no |  | 6 | **2** | 0 | 0 | 0 | 0 | 0 |
| 155 | yes | yes | yes |  | 23 | **2** | 0 | 0 | 0 | 0 | 0 |
| 160 | yes | yes | no |  | 5 | **2** | 0 | 0 | 0 | 0 | 0 |
| 161 | yes | yes | no |  | 7 | **2** | 0 | 0 | 0 | 0 | 0 |
| 162 | yes | yes | no |  | 15 | **2** | 0 | 2 | 0 | 0 | 2 |
| 155 | yes | yes | yes |  | 23 | **2** | 0 | 0 | 0 | 0 | 0 |
| 160 | yes | yes | no |  | 5 | **2** | 0 | 0 | 0 | 0 | 0 |
| **12 to 17 years of age** | | | | | | | | | | | |
| 87 | yes | yes | no |  | 2 | **1** | 0 | 0 | 0 | 0 | 0 |
| 92 | yes | yes | no |  | 3 | **1** | 0 | 0 | 0 | 0 | 0 |
| 101 | yes | yes | yes |  | 3 | **1** | 0 | 0 | 0 | 0 | 0 |
| 139 | yes | yes | no |  | 2 | **1** | 0 | 0 | 0 | 0 | 0 |
| 170 | yes | yes | no |  | 1 | **1** | 0 | 1 | 0 | 0 | 1 |
|  | | | | | | | | | | | |
| 6 | yes | yes | yes | no | 14 | **2** | 0 | 0 | 0 | 0 | 0 |
| 57 | yes | yes | no |  | 32 | **2** | 0 | 1 | 0 | 0 | 1 |
| 61 | yes | yes | no |  | 37 | **2** | 0 | 2 | 0 | 0 | 2 |
| 66 | yes | yes | yes |  | 10 | **2** | 0 | 0 | 0 | 0 | 0 |
| 95 | yes | yes | yes |  | 8 | **2** | 0 | 2 | 2 | 0 | 0 |
| 96 | yes | yes | yes |  | 37 | **2** | 0 | 0 | 0 | 0 | 0 |
| 104 | yes | yes | yes |  | 5 | **2** | 0 | 0 | 0 | 0 | 0 |
| 113 | yes | yes | yes |  | 9 | **2** | 0 | 0 | 0 | 0 | 0 |
| 122 | yes | yes | yes |  | 12 | **2** | 0 | 0 | 0 | 0 | 0 |
| 129 | yes | yes | no |  | 11 | **2** | 0 | 2 | 0 | 0 | 2 |
| 136 | yes | yes | no |  | 40 | **2** | 0 | 0 | 0 | 0 | 0 |
| 143 | yes | yes | no |  | 5 | **2** | 0 | 1 | 0 | 0 | 1 |
| 154 | yes | yes | no |  | 36 | **2** | 0 | 0 | 0 | 0 | 0 |
| **Below 12 years of age** | | | | | | | | | | | |
| 34 | yes | yes | yes | no | 3 | **1** | 0 | 0 | 0 | 0 | 0 |
| 39 | yes | yes | yes | no | 1 | **1** | 0 | 0 | 0 | 0 | 0 |
| 43 | yes | yes | no | no | 0 | **1** | 0 | 2 | 0 | 0 | 2 |
| 63 | yes | yes | no |  | 4 | **1** | 0 | 2 | 0 | 0 | 2 |
| 72 | yes | yes | yes |  | 4 | **1** | 0 | 1 | 0 | 0 | 1 |
| 77 | yes | yes | no |  | 3 | **1** | 0 | 2 | 0 | 0 | 2 |
| 79 | yes | yes | yes |  | 2 | **1** | 0 | 2 | 0 | 0 | 2 |
| 82 | yes | yes | no |  | 3 | **1** | 0 | 1 | 0 | 0 | 1 |
| 89 | yes | yes | no |  | 1 | **1** | 0 | 1 | 0 | 0 | 1 |
| 90 | yes | yes | no |  | 4 | **1** | 0 | 0 | 0 | 0 | 0 |
| 102 | yes | yes | yes |  | 1 | **1** | 0 | 0 | 0 | 0 | 0 |
| 105 | yes | yes | no |  | 4 | **1** | 0 | 0 | 0 | 0 | 0 |
| 106 | yes | yes | yes |  | 3 | **1** | 0 | 0 | 0 | 0 | 0 |
| 117 | yes | yes | no |  | 2 | **1** | 0 | 0 | 0 | 0 | 0 |
| 118 | yes | yes | yes |  | 4 | **1** | 0 | 2 | 0 | 0 | 2 |
| 121 | yes | yes | no |  | 3 | **1** | 0 | 0 | 0 | 0 | 0 |
| 128 | yes | yes | no |  | 2 | **1** | 0 | 2 | 0 | 0 | 2 |
| 156 | yes | yes | no |  | 3 | **1** | 0 | 0 | 0 | 0 | 0 |
| 157 | yes | yes | yes |  | 4 | **1** | 0 | 0 | 0 | 0 | 0 |
| 158 | yes | yes | no |  | 2 | **1** | 0 | 0 | 0 | 0 | 0 |
| 164 | yes | yes | no |  | 1 | **1** | 0 | 0 | 0 | 0 | 0 |
|  | | | | | | | | | | | |
| 3 | yes |  | yes | no | 21 | **2** | 0 | 0 | 0 | 0 | 0 |
| 26 | yes | yes | yes | no | 132 | **2** | 0 | 1 | 0 | 0 | 1 |
| 47 | yes | yes | no | no | 131 | **2** | 0 | 0 | 0 | 0 | 0 |
| 73 | yes | yes | yes |  | 13 | **2** | 0 | 0 | 0 | 0 | 0 |
| 81 | yes | yes | no |  | 26 | **2** | 0 | 0 | 0 | 0 | 0 |
| 86 | yes | yes | yes |  | 8 | **2** | 0 | 1 | 0 | 0 | 1 |
| 99 | yes | yes | yes |  | 15 | **2** | 0 | 0 | 0 | 0 | 0 |
| 107 | yes | yes | no | yes | 5 | **2** | 0 | 0 | 0 | 0 | 0 |
| 125 | yes | yes | no |  | 5 | **2** | 0 | 0 | 0 | 0 | 0 |
| 126 | yes | yes | yes |  | 10 | **2** | 0 | 0 | 0 | 0 | 0 |
| 132 | yes | yes | yes |  | 5 | **2** | 0 | 0 | 0 | 0 | 0 |
| 163 | yes | yes | yes |  | 18 | **2** | 0 | 0 | 0 | 0 | 0 |
|  | | | | | | | | | | | |
| **B: Data for patients who received trypanocides for prior HAT** | | | | | | | | | | | |
| **Patient** | **Serology** | | | | | **Stage** | **LAMP Assay Results*** | | | | |
|  | **CATT Positive** | **Trypanosomes in** | | | **Number**  **of cells**  **In CSF** |  | **RIME LAMP** | | | **TBG1 LAMP** | |
|  |  |  |  |  |  |  | **Preincubation** | | | **Preincubation** | |
|  |  | **Blood** | **Lymph** | **CSF** |  |  | **Sham**  **60 min** | **Tx100**  **60 min** | **Tx100**  **0 min** | **Sham**  **60 min** | **Tx100**  **60 min** |
| **Adults** | | | | | | | | | | | |
| 2 | yes | no | yes | no | 1 | **1** | 2 | 2 | 2 | ND | 2 |
| 52 | yes | yes | yes | no | 1 | **1** | 0 | 2 | 0 | 0 | 2 |
| 56 | yes | yes | yes |  | 0 | **1** | 0 | 1 | 0 | 0 | 1 |
| 58 | yes | yes | no |  | 0 | **1** | 0 | 2 | 0 | 0 | 2 |
| 62 | yes | yes | no |  | 0 | **1** | 0 | 2 | 0 | 0 | 2 |
| 80 | yes | yes | no |  | 3 | **1** | 0 | 2 | 0 | 0 | 2 |
| 133 | yes | yes | yes |  | 0 | **1** | 0 | 1 | 0 | 0 | 1 |
|  | | | | | | | | | | | |
| 10 | yes | yes | yes | no | 5 | **2** | 0 | 2 | 0 | 0 | 2 |
| 20 | yes | yes | yes | yes | 190 | **2** | 0 | 2 | 0 | 0 | 2 |
| 23 | yes | yes | yes | no | 39 | **2** | 0 | 0 | 0 | 0 | 0 |
| 38 | yes | yes | no | no | 39 | **2** | 0 | 2 | 0 | 0 | 2 |
| 54 | yes | yes | yes | no | 10 | **2** | 0 | 1 | 0 | 0 | 1 |
| 70 | yes | yes | no |  | 9 | **2** | 0 | 0 | 0 | 0 | 0 |
| 84 | yes | yes | yes |  | 5 | **2** | 0 | 0 | 0 | 0 | 0 |
| 134 | yes | yes | yes |  | 16 | **2** | 0 | 2 | 0 | 0 | 2 |
| 144 | yes | yes | yes |  | 49 | **2** | 0 | 0 | 0 | 0 | 0 |
| 153 | yes | yes | yes |  | 100 | **2** | 0 | 0 | 0 | 0 | 0 |
| **12 to 17 years of age** | | | | | | | | | | | |
| 55 | yes | yes | no |  | 0 | **1** | 0 | 1 | 0 | 0 | 1 |
|  | | | | | | | | | | | |
| 71 | yes | yes | yes |  | 12 | **2** | 0 | 0 | 0 | 0 | 0 |
| 93 | yes | yes | no |  | 56 | **2** | 0 | 0 | 0 | 0 | 0 |
| **Below 12 years of age** | | | | | | | | | | | |
| 25 | yes | yes | yes | no | 2 | **1** | 0 | 0 | 0 | 0 | 0 |
|  | | | | | | | | | | | |
| 17 | yes | no | no | no | 125 | **2** | 0 | 0 | 0 | 0 | 0 |
|  | | | | | | | | | | | |
| *** Legend:** Sham or Tx100 pretreated (0 or 60 min) CSF samples (I μL) were assayed in duplicate and scored as follows:  **0** = Both samples negative  **1** = 1 out of 2 samples positive  **2** = Both samples positive  **ND** = Not done due to lack of sample | | | | | | | | | | | |
