# Supplementary material for: Using detergent-enhanced LAMP for African trypanosome detection in human cerebrospinal fluid and implications for disease staging
Source: PLoS Negl Trop Dis. 2019 Aug 19;13(8):e0007631. doi: 10.1371/journal.pntd.0007631 (PMC6715242; doi:10.1371/journal.pntd.0007631)
Supplement: S2 Table — (DOCX) [file pntd.0007631.s003.docx]

| **S2 Table: The LAMP Primer Sets.** | | | |
| --- | --- | --- | --- |
| **Primer Set**  (Specificity) | **Target**  **Gene** | **Primer**  **Sequences (5’-3’)** | **Ref** |
| **RIME LAMP**  (Pan-*T. brucei*) | **RIME** | F3: CTGTCCGGTGATGTGGAAC  B3: CGTGCCTTCGTGAGAGTTTC  FIP: GGAATACAGCAGATGGGGCGAGGCCAATTGGCATCTTTGGGA  BIP: AAGGGAGACTCTGCCACAGTCGTCAGCCATCACCGTAGAGC  LF: GCCTCCCACCCTGGACTC  LB: AGACCGATAGCATCTCAG | [20] |
| **TBG1 LAMP**  (*T.b. gambiense*) | **5.8S-ITS2** | F3: AAGCTCTCTCGAGCCATC  B3: TTTTGGCTCCGTTGGTCG  FIP: AAGCGCTGAGATGTTGCTGTGGAAGCGGAAGCAAGAATGACC  BIP: TATGCCGCGGCGTCAAACATAACGCTATTGGCGCAAG | [29] |
| **Legend:** Outer forward (**F3**), outer backward (**B3**), forward inner (**FIP**), backward inner (**BIP**) primers,  loop forward (**LF**) and loop backward (**LB**) primers. | | |  |
